# 1606. Dual Trajectories of Antiretroviral Therapy Adherence and Polypharmacy in Women with HIV in the United Sate

**DOI:** 10.1093/ofid/ofad500.1441

**Published:** 2023-11-27

**Authors:** A B U B A K E R SAEED, Musie Ghebremichael, Deborah Konkle-Parker, Deborah Jones Weiss, Shelby Collins, Adaora A Adimora, Michael F Schneider, Mardge H Cohen, Bani Tamraz, Michael Plankey, Tracey Wilson, Adebola adedimeji, Jessica Haberer, Denise L Jacobson Jacobson

**Affiliations:** Center for Global Health, Massachusetts General Hospital, Boston, Massachusetts; The Ragon Institute of MGH, MIT and Harvard, Cambridge, Massachusetts; University of Mississippi Medical Center, Jackson, MS; University of Miami Miller School of Medicine, Miami, Florida; Emory University School of Medicine, Division of Infectious Disease, Atlanta, Georgia; Department of Medicine, University of North Carolina at Chapel Hill, Chapel Hill, North Carolina; Department of Epidemiology, Johns Hopkins Bloomberg School of Public Health, Baltimore, Maryland; Department of Medicine, Stroger Hospital of Cook County, Chicago, Illinois; University of California, San Francisco, School of Pharmacy, San Francisco, California; Georgetown University, Washington, District of Columbia; Downstate Health Sciences University, Brooklyn, New York; Albert Einstein College of Medicine, Bronx, New York; Harvard Medical School, Boston, MA; Center for Biostatistics in AIDS Research, Department of Epidemiology, Harvard T. H. Chan School of Public Health, Boston, Massachusetts

## Abstract

**Background:**

Polypharmacy using five or more medications with antiretroviral therapy (ART) may increase the risk of nonadherence to prescribed treatment. We aimed to identify the interrelationship between trajectories of adherence to ART and polypharmacy.

**Methods:**

We included women with HIV (aged >18 years) enrolled in the Women's Interagency HIV Study in the United States from 2014-2019. We used group-based trajectory modeling (GBTM) to identify trajectories of adherence to ART and polypharmacy and dual GBTM to identify the interrelationship between adherence and polypharmacy.

**Results:**

Overall, 1538 were eligible (median age of 49 years). GBTM revealed five latent trajectories of adherence with 42% of women grouped in the consistently moderate trajectory. GBTM identified four polypharmacy trajectories with 45% categorized in the consistently low group.

**Conclusion:**

The joint model did not reveal any interrelationship between ART adherence and polypharmacy trajectories. Future research should consider examining the interrelationship between both variables using objective measures of adherence.

**Disclosures:**

**Jessica Haberer, MD, MS**, Merck: Advisor/Consultant

